# Coexisting high-grade glandular and squamous cervical lesions and human papillomavirus infections

**DOI:** 10.1038/sj.bjc.6601204

**Published:** 2003-08-26

**Authors:** R L M Bekkers, J Bulten, A Wiersma-van Tilburg, M Mravunac, C P T Schijf, L F A G Massuger, W G V Quint, W J G Melchers

**Affiliations:** 1 Department of Gynecology/Obstetrics, University Medical Center Nijmegen, St Radboud, PO Box 9101, 6500 HB Nijmegen, The Netherlands; 2 Department of Pathology, University Medical Center Nijmegen, St Radboud, PO Box 9101, 6500 HB Nijmegen, The Netherlands; 3 Department of Pathology, Rijnstate Hospital Arnhem, Arnhem, The Netherlands; 4 Department of Pathology, Canisius Wilhelmina Hospital Nijmegen, Nijmegen, The Netherlands; 5 Delft Diagnostic Laboratory, Delft, The Netherlands; 6 Department of Medical Microbiology, University Medical Center Nijmegen, St Radboud, PO Box 9101, 6500 HB Nijmegen, The Netherlands

**Keywords:** ACIS, Coexisting CIN, HPV genotypes

## Abstract

The frequency of high-risk human papillomavirus (hr-HPV) genotypes in patients with adenocarcinoma *in situ* (ACIS) with coexisting cervical intraepithelial neoplasia (CIN), ACIS without coexisting CIN, and high-grade CIN (CIN II/III) was studied, in order to gain more insight into the relation between hr-HPV infections and the development of coexisting squamous and glandular lesions. The SPF_10_ LiPA PCR was used to detect simultaneously 25 different HPV genotypes in biopsies obtained from 90 patients with CIN II/III, 47 patients with ACIS without coexisting CIN, and 49 patients with ACIS and coexisting CIN. hr-HPV was detected in 84 patients (93%) with CIN II/III, 38 patients (81%) with ACIS without CIN, and in 47 patients (96%) with ACIS and coexisting CIN. A total of 13 different hr-HPV genotypes were detected in patients with CIN II/III, and only five in patients with ACIS with/without coexisting CIN. HPV 31, multiple hr-HPV genotypes, and HPV genotypes other than 16, 18, and 45 were significantly more often detected in patients with CIN II/III, while HPV 18 was significantly more often detected in patients with ACIS with/without CIN. There were no significant differences in the frequency of specific hr-HPV genotypes between patients with ACIS with or without coexisting CIN. In conclusion, the frequency of specific hr-HPV genotypes is similar for patients with ACIS without CIN and patients with ACIS and coexisting CIN, but is significantly different for patients with CIN II/III without ACIS. These findings suggest that squamous lesions, coexisting with high-grade glandular lesions, are aetiologically different from squamous lesions without coexisting glandular lesions.

A causal relation between high-risk human papillomavirus (hr-HPV) infections and cervical cancer has been documented in the literature beyond reasonable doubt (Bosch *et al*, 2002; Wright *et al*, 2002). High-risk human papillomavirus can be detected in almost 100% of squamous carcinomas and adenocarcinomas of the uterine cervix (Walboomers *et al*, 1999; Pirog *et al*, 2000). Numerous studies have suggested that the development of squamous cervical carcinoma is preceded by cervical intraepithelial neoplasia (CIN) (Wright *et al*, 2002; Bosch *et al*, 2002).

The frequency of hr-HPV genotypes in CIN lesions has been studied previously and its detection rate rises with increasing severity of the CIN lesion (Bekkers *et al*, 2002a; Bosch *et al*, 2002; Wright *et al*, 2002). Only limited studies are available on the frequency of hr-HPV genotypes in premalignant glandular lesions and the number of patients in most studies is rather low (see Table 1

**Table 1:** Summary of all studies since 1990 with more than 20 ACIS patients in relation with hr-HPV and/or coexisting CIN

**Author and year**	**Patients (*n*)**	**hr-HPV pos**	**HPV 16**	**HPV 18**	**Coexisting CIN**
Riethdorf *et al* (2002)	42	32	22	10	ND
		(76%)	(53%)	(24%)	
Madeleine *et al* (2001)	82	71	32	43	ND
		(87%)	(39%)	(52%)	
Riethdorf *et al* (2000)	33	29	21	8	14
		(88%)	(64%)	(24%)	(42%)
Pirog *et al* (2000)	23	23	10	6	9
		(100%)	(43%)	(26%)	(39%)
Anciaux *et al* (1997)	21	16	6	10	9
		(76%)	(29%)	(47%)	(43%)
Duggan *et al* (1994)	37	23	8	15	ND
		(66%)	(23%)	(43%)	
Higgins *et al* (1992)	42	39	13	28	22
		(95%)	(31%)	(67%)	(52%)
Leary *et al* (1991)	30	21	10	11	15
		(70%)	(33%)	(37%)	(50%)
Bekkers *et al* (present study)	96	85	39	41	49
		(89%)	(41%)	(43%)	(51%)

ACIS=adenocarcinoma *in situ*; hr-HPV= high-risk human papillomavirus; CIN=cervical intraepithelial neoplasia; HPV=human papillomavirus; ND=not documented.

) (Colgan and Lickrish, 1990; Leary *et al*, 1991; Higgins *et al*, 1992; Duggan *et al*, 1995; Anciaux *et al*, 1997; McLachlin *et al*, 1997; Pirog *et al*, 2000; Riethdorf *et al*, 2000,^2002^; Madeleine *et al*, 2001). High-risk human papillomavirus was detected in 66–100% of patients with ACIS (Anciaux *et al*, 1997; Riethdorf *et al*, 2000). Approximately 48% of all women diagnosed with ACIS have coexisting squamous lesions (Colgan and Lickrish, 1990; Leary *et al*, 1991; Higgins *et al*, 1992; Duggan *et al*, 1995; Anciaux *et al*, 1997; McLachlin *et al*, 1997; Pirog *et al*, 2000; Riethdorf *et al*, 2000,^2002^; Madeleine *et al*, 2001), but the frequency of specific hr-HPV genotypes in patients with ACIS has not been studied in relation with the presence or absence of coexisting CIN lesions. In this study, the frequency of specific hr-HPV genotypes in patients with ACIS and coexisting CIN is compared with its frequency in patients with ACIS without coexisting CIN, and patients with CIN without ACIS, in order to gain more insight into the relation between hr-HPV infections and the development of coexisting squamous and glandular lesions.

## MATERIAL AND METHODS

### Glandular lesions

In the automated databases of the pathology laboratories of the University Medical Center Nijmegen, the Canisius Wilhelmina Hospital Nijmegen, and the Rijnstate Hospital Arnhem, 120 patients diagnosed with ACIS between 1988 and 2000 were identified. No histopathological material was left for further study of nine patients, and six patients had a histopathological diagnosis of invasive carcinoma within 1 month of the diagnosis of ACIS. Of the remaining 105 patients, the histopathological slides of a biopsy-, cone-, or hysterectomy specimen were re-examined independently, by three experienced gynaecopathologists (JB, AWvT, and MM). Complete agreement on the diagnosis of ACIS was reached in 96 patients (91%), according to the criteria of Brown and Wells. The criteria of Brown and Wells take architectural (glandular irregularity and complexity) and cytological criteria (nuclear enlargement, hyperchromasia, pseudostratification of nuclei, increased or abnormal mitoses) into account (Wells and Brown, 1986). The three pathologists reached no consensus on the diagnosis of ACIS in nine patients, and these patients were excluded.

Coexisting squamous intraepithelial lesions were detected and confirmed by consensus of the three pathologists (JB, AWvT, MM) in the slides of 49 of the 96 patients (51%); 35 patients (71%) had coexisting CIN 3 (severe dysplasia, or carcinoma *in situ*), eight patients (16%) had coexisting CIN 2 (moderate dysplasia), and six patients (12%) had coexisting CIN 1 (mild dysplasia). In 41 of these 49 patients (84%), CIN and ACIS were diagnosed in the same slide/section, and hr-HPV detection was carried out on adjacent sections, containing both the squamous and glandular lesion. In eight patients, CIN was diagnosed in a different slide than in which ACIS was diagnosed and on which hr-HPV detection was performed. Five of these eight patients had coexisting CIN I, two had coexisting CIN II, and one had coexisting CIN III. The mean age of the 96 patients was 38.3 years (28–73) at the time of the diagnosis.

A cervical scrape preceding the diagnosis of ACIS less than 6 months was made in 43 of the 49 patients (88%) with ACIS and coexisting CIN, and in 40 of the 47 patients (85%) with ACIS without coexisting CIN. The cervical scrape indicated a high-grade glandular (moderate atypia–ACIS), a high-grade squamous lesion (moderate dysplasia–CIS), or both in, respectively, 13 (30%), 15 (35%), and 13 (30%) patients with ACIS and coexisting CIN, and in, respectively, 24 (60%), four (10%), and eight (20%) patients with ACIS without coexisting CIN. The remaining six patients had only mild atypia of glandular cells in the cervical scrape.

### Squamous lesions

A control group of 90 patients consisted of patients who consulted the colposcopy clinic at the UMC between 1997 and 1999 and who were diagnosed with high-grade CIN in the biopsy specimen of a large-loop excision of the transformation zone. All slides were reviewed by an experienced gynaecopathologist (JB), and the diagnosis of high-grade CIN was confirmed. Of these 90 patients, 18 patients (20%) were diagnosed with moderate dysplasia (CIN II), 36 patients (40%) with severe dysplasia (CIN III), and 36 patients (40%) with carcinoma *in situ* (CIN III). The mean age at the time of diagnosis was 37.6 years (28–59).

A cervical scrape preceding the diagnosis of CIN II/III less than 6 months was taken of all 90 patients. The cervical scrape indicated a high-grade squamous lesion in 80 patients (89%), a high-grade glandular and squamous lesion in one patient (1%), and low-grade squamous lesions in nine patients (10%).

Taking the preceding cervical scrapes of all patients with ACIS and CIN II/III together, the cervical scrape indicated a high-grade glandular lesion in 37 patients (21%), a high-grade squamous lesion in 99 patients (57%), both in 22 patients (13%), and only low-grade lesions in 15 patients (9%).

### HPV detection

A 3-*μ*m section of the histopathological specimens, on which ACIS and/or CIN II/III was diagnosed, was taken for HPV analysis. In 41 of the 49 patients with ACIS and coexisting CIN, HPV detection was carried out on a section containing both the glandular and squamous lesion. HPV detection was performed using a broad-spectrum short-fragment polymerase chain reaction (SPF_10_ PCR) as previously described (Kleter *et al*, 1998,^1999^; Melchers *et al*, 1999; Quint *et al*, 2001; Bekkers *et al*, 2002b). In case of a positive PCR, subsequent genotyping was performed via a reverse hybridisation line probe assay (LiPA), allowing for simultaneous typing of 25 HPV genotypes, including hr-HPV genotypes 16, 18, 31, 33, 35, 39, 45, 51, 52, 56, 58, 59, 66, and 68. This SPF_10_ LiPA PCR HPV detection method is highly sensitive, specific and reproducible and has been clinically validated (Kleter *et al*, 1998,^1999^; Melchers *et al*, 1999; Pirog *et al*, 2000; Quint *et al*, 2001). If more than one hr-HPV genotype was detected with the SPF_10_ LiPA PCR in a single sample of an individual patient, that patient was considered to be infected with multiple hr-HPV genotypes.

### Analysis

The frequency of specific hr-HPV genotypes in patients with ACIS without coexisting CIN, ACIS with coexisting CIN, and CIN II/III without ACIS were compared. The frequency of specific hr-HPV genotypes was also investigated in relation with the suspicion of a high-grade glandular and/or high-grade squamous lesion in the preceding cervical scrape. Statistical analysis including *χ*^2^ tests and independent *t*-tests was performed, considering all values of *P*<0.05 to be significant.

## RESULTS

**Figure 1:**
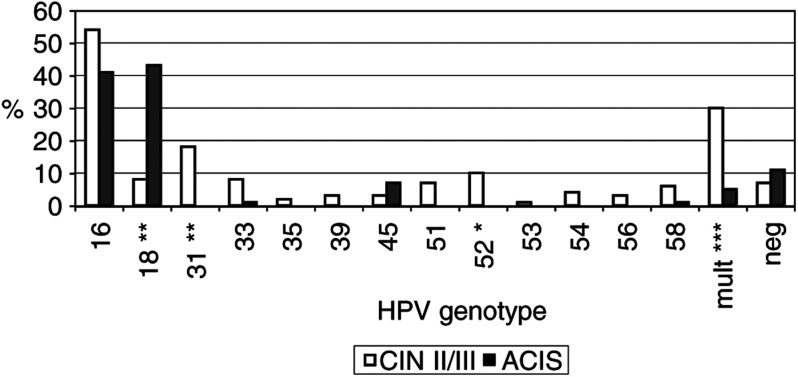
Relative frequency of specific hr-HPV genotypes in patients with CIN II/III (90 patients) and patients with ACIS (96 patients). ^*^P<0.05, ^**^P<0.01, ^***^P<0.001.

High-risk human papillomavirus genotypes were detected in 93% of the patients with CIN II/III, 81% of the patients with ACIS without coexisting CIN, and in 96% of the patients with ACIS and coexisting CIN. Figure 1

shows that HPV 31 and multiple hr-HPV genotypes were significantly more often detected in patients with CIN II/III, while HPV 18 was significantly more often detected in patients with ACIS (with or without coexisting CIN). Among patients with CIN II/III, 13 different hr-HPV genotypes were detected, compared with only five different hr-HPV genotypes in patients with ACIS with or without coexisting CIN.

HPV 16 was the most frequently detected HPV genotype in patients with CIN II/III (54%), while both HPV 16 and 18 showed a high frequency of, respectively, 41 and 43% in all patients with ACIS (see Figure 1). HPV 16 or 18 were present in 79% of all patients with ACIS (five patients had a double infection with HPV 16 and 18), and in 61% of the patients with CIN II/III (one patient had a double infection with HPV 16 and 18).

Table 2

**Table 2:** Frequency of hr-HPV genotypes in patients with CIN II/III compared with patients with ACIS and coexisting CIN

**hr-HPV genotype**	**CIN II/III *n*=90**	**ACIS with CIN *n*=49**	**Significance**
16	49 (54%)	19 (39%)	NS
18	7 (8%)	23 (47%)	*χ*^2^=26.49, *P*<0.001
31	15 (17%)	0 (0%)	*χ*^2^=8.38, *P*<0.01
45	3 (3%)	5 (10%)	NS
Other	34 (38%)	2 (4%)	*χ*^2^=19.73, *P*<0.001
Multiple	26 (29%)	2 (4%)	*χ*^2^=12.48, *P*<0.001
Total hr-HPV positive	84 (93%)	47 (96%)	NS
hr-HPV negative	6 (7%)	2 (4%)	NS

ACIS=adenocarcinoma *in situ*; hr-HPV= high-risk human papillomavirus; CIN=cervical intraepithelial neoplasia; NS=not significant.

shows that there were no significant differences in the frequency of HPV 16, 45, and hr-HPV-negative patients between patients with CIN II/III and patients with ACIS and coexisting CIN. The frequency of HPV 18 was significantly lower, and the frequency of HPV 31, multiple hr-HPV, and hr-HPV genotypes other than HPV 16, 18, 31, and 45 was significantly higher in patients with CIN II/III compared with patients with ACIS and coexisting CIN. This means that the frequency of HPV 18 is significantly higher in CIN lesions coexisting with ACIS than in solitary CIN lesions.

Table 3

**Table 3:** Frequency of hr-HPV genotypes in patients with ACIS with coexisting CIN compared with patients with ACIS without coexisting CIN

**hr-HPV genotype**	**ACIS with CIN *n*=49**	**ACIS without CIN *n*=47**	**Significance**
16	19 (39%)	20 (43%)	NS
18	23 (47%)	18 (38%)	NS
31	0 (0%)	0 (0%)	NS
45	5 (10%)	2 (4%)	NS
Other	2 (4%)	1 (2%)	NS
Multiple	2 (4%)	3 (6%)	NS
Total hr-HPV positive	47 (96%)	38 (81%)	*χ*^2^=6.07, *P*<0.02
hr-HPV negative	2 (4%)	9 (19%)	*χ*^2^=6.63, *P*<0.01

ACIS=adenocarcinoma *in situ*; hr-HPV= high-risk human papillomavirus; CIN=cervical intraepithelial neoplasia; NS=not significant.

shows that there were no significant differences in the frequency of HPV 16, 18, 31, 45, multiple hr-HPV, and other hr-HPV genotypes between patients with ACIS without coexisting CIN and patients with ACIS and coexisting CIN. The number of hr-HPV-negative patients was significantly higher in patients with ACIS without coexisting CIN.

Among the patients with ACIS and coexisting CIN, there were no significant differences in the frequency of specific hr-HPV genotypes regarding the different degrees of coexisting CIN.

The mean age of the patients with ACIS without CIN was 40.1 years, while the mean age of patients with ACIS and coexisting CIN was 36.3 years (*t*=2.32, *P*<0.05). There was no difference between the mean age of the total group of patients with ACIS and the patients with CIN II/III (38.2 and 37.6 years, respectively).

Within the group of patients with CIN II/III, there were no significant differences in the frequency of HPV 16, 18, 31, and 45, multiple hr-HPV, other hr-HPV genotypes, or hr-HPV-negative patients between patients with moderate dysplasia, severe dysplasia, or carcinoma *in situ*. Multiple hr-HPV genotypes were detected in, respectively, 22, 28, and 33% of these patients.

High-risk human papillomavirus was detected in 33 of the 37 patients (89%) with a cervical scrape that indicated a high-grade glandular lesion, in 92 of the 99 patients (93%) with a cervical scrape that indicated a high-grade squamous lesion, in all 22 patients (100%) with a cervical scrape that indicated both a high-grade glandular and squamous lesion, and in 13 of the 15 patients (87%) with a cervical scrape that indicated only a low-grade lesion. There were no significant differences in the frequency of HPV 16, 18, 31, 45, multiple or other HPV genotypes regarding the presence or absence of a suspicion of a high-grade squamous and/or glandular lesion in the cervical scrapes.

## DISCUSSION

The number of patients with ACIS and coexisting CIN lesions among all patients with ACIS in this study is similar to the numbers reported in the literature (Table 1). No significant differences in the frequency of different hr-HPV genotypes were observed between patients with ACIS with coexisting CIN, and patients with ACIS without coexisting CIN, as has been reported previously (Anciaux *et al*, 1997; Riethdorf *et al*, 2000; Zaino, 2002). However, significant differences in the frequency of HPV 18, HPV 31, multiple hr-HPV genotypes, and hr-HPV genotypes other than HPV 16, 18, 31, and 45 were observed between patients with CIN II/III and patients with ACIS, either with or without coexisting CIN. One may hypothesise that coexisting glandular and squamous lesions share a common aetiology, different from solitary squamous lesions, under the influence of specific hr-HPV genotypes, as has been previously described for squamous lesions (Burghardt and Ostor, 1983; Park *et al*, 1998; Bekkers *et al*, 2002b). Indeed, in 41 of the 49 patients (84%) in the present study the ACIS lesion was adjacent to the coexisting squamous lesion. Furthermore, Colgan and Lickrish (1990) found no differences in the linear extend, circumferential extend, multifocality, or the co-presence of invasive adenocarcinoma between patients with ACIS without CIN and ACIS with coexisting CIN lesions. These observations seem to support the hypothesis that ACIS and coexisting CIN lesions share a common aetiology.

Only five different hr-HPV genotypes (HPV 16, 18, 33, 45, 58) were detected in patients with ACIS (of which HPV 33 and 58 in <2% of the patients), compared with 13 different hr-HPV genotypes in patients with CIN II/III. Other authors also found a limited number of hr-HPV genotypes (three to seven) among patients with ACIS (Pirog *et al*, 2000; Madeleine *et al*, 2001). This may suggest that only certain hr-HPV genotypes are able to infect and/or induce lesions in the glandular mucosa. This fact may be the reason for the significantly lower detection rate of multiple hr-HPV genotypes, and the significantly higher detection rate of HPV 18 in the cervix of patients with ACIS. However, higher rates (13–22%) of multiple hr-HPV infections among patients with ACIS have been described in the literature (Pirog *et al*, 2000; Madeleine *et al*, 2001). The lower rate of multiple hr-HPV genotypes in the present study is probably the result of the very restrictive diagnostic criteria, and the consensus agreement by three pathologists, that was used to diagnose ACIS.

The high frequency of a single hr-HPV genotype in patients with ACIS compared with patients with CIN II/III may reflect the monoclonal aspect of the ACIS lesion, as is often the case in invasive carcinomas (Walboomers *et al*, 1999). Patients with (squamous) CIS in this study showed a higher prevalence (33%) of multiple hr-HPV genotypes, as well as a higher number of different hr-HPV genotypes (12), which may be regarded as another indication that ACIS lesions may have a biologic behaviour and/or aetiology that is different from squamous *in situ* lesions (Schoolland *et al*, 2002).

The number of hr-HPV-negative patients with ACIS without CIN in this study was significantly higher than in patients with ACIS and coexisting CIN. ACIS may be a precursor of different subtypes of invasive adenocarcinoma (like small cell or endometrioid adenocarcinoma) that have a lower or no prevalence of hr-HPV (Lee, 1999; Pirog *et al*, 2000; Schoolland *et al*, 2002). These subtypes are rare, and distinction on a biopsy with only ACIS proved to be difficult (Lee, 1999; Schoolland *et al*, 2002). It is possible that these hr-HPV-negative subtypes of ACIS are less often associated with coexisting CIN, resulting in a higher rate of hr-HPV-negative patients among patients with ACIS without CIN.

The significant difference in age between patients with ACIS without CIN and patients with ACIS with coexisting CIN, in which the latter are younger, has been described previously (Colgan and Lickrish, 1990; Pirog *et al*, 2000). Different explanations for this age difference are possible. Firstly, the lesions in patients with ACIS and coexisting CIN may show a faster progression, leading to detection at a younger age. This has been described previously for HPV 18-related lesions (Barnes *et al*, 1988), but we did not find a difference in HPV 18 prevalence between patients with ACIS alone or ACIS and coexisting CIN. Secondly, the involvement of the squamous epithelium may be the reason for the detection of ACIS with coexisting CIN at a younger age, since ecto-cervical lesions are more easily detected. Thirdly, it may be a coincidental finding especially since we did not find a difference in mean age between patients with CIN II/III and the total group of patients with ACIS.

There was no significant difference in the overall sensitivity of the preceding cervical scrape for the detection of either ACIS or CIN II/III in the present study (respectively 93 and 90%). The observed sensitivity to detect ACIS was higher than reported in the literature (Lee, 1999; Schoolland *et al*, 2002). However, these studies used cervical scrapes from a cervical cancer screening programme, while in the present study several patients had more than one preceding cervical scrape taken, which may have alerted the pathologist, leading to the higher sensitivity.

We did not find a relation between the presence of certain hr-HPV genotypes in the biopsy, and the sensitivity of the cervical scrape in detecting ACIS and/or CIN II/III lesions. This indicates that the detection of a lesion in the cervical scrape is not influenced by the genotype of hr-HPV infecting that lesion.

In conclusion, patients with ACIS have significantly more often HPV 18 infections, while patients with CIN II/III have significantly more often infections with HPV 31, HPV genotypes other than 16, 18, 31, and 45, and multiple hr-HPV genotypes. The detection of high-grade glandular and/or squamous lesion by cervical scrapes is not influenced by the hr-HPV genotype associated with the lesion. Among patients with ACIS, patients with coexisting CIN lesions tend to be younger, but they have a similar frequency of different hr-HPV genotypes than patients with ACIS without CIN, while the frequency of specific hr-HPV genotypes differs significantly from those of patients with CIN II/III without ACIS. These findings suggest that squamous lesions, coexisting with high-grade glandular lesions, are aetiologically different from squamous lesions without coexisting glandular lesions. The clinical implication of these findings needs further study.
